# Functional acute acquired comitant esotropia: clinical characteristics and efficacy of single Botulinum toxin type A injection

**DOI:** 10.1186/s12886-020-01739-9

**Published:** 2020-11-25

**Authors:** Luyao Tong, Xiaoning Yu, Xiajing Tang, Yidong Zhang, Sifan Zheng, Zhaohui Sun

**Affiliations:** 1 Eye Center of the Second Affiliated Hospital, School of Medicine, Zhejiang University, Jiefang Road 88#, Hangzhou, 310009 Zhejiang Province China; 2 Affiliated Hospital of Medical School of Ningbo University, Ningbo, Zhejiang Province China; 3 GKT School of Medical Education, King’s College London, London, SE1 1UL England

**Keywords:** Acute acquired comitant esotropia, Botulinum toxin type a, Binocular visual function

## Abstract

**Background:**

To examine the clinical features of acute acquired comitant esotropia (AACE) and to evaluate the clinical effectiveness of a single injection of botulinum toxin type A (BTXA) on binocular visual function (BVF).

**Methods:**

This retrospective, observational case series study enrolled patients with AACE examined from October 2018–May 2019. BTXA was injected into the both medial rectus muscles. The refractive error, best-corrected visual acuity (BCVA), stereoacuity, vergence, accommodation, the horizontal angle of deviation, and the gradient accommodative convergence/accommodation (AC/A) ratio were measured pre- and post-BTXA injection. Data pre- and postinjection were compared by the Wilcoxon signed-rank test. A Spearman correlation coefficient was calculated to explore the relationships between demographic characteristics and BVF.

**Results:**

Twenty-two AACE cases were included. Compared with preinjection deviation, the postinjection deviation in the primary position was smaller for near (*p* < 0.001) and distance (*p* < 0.001) fixation at 3 months after injection (BTXA). Furthermore, convergence was better for near (*p* = 0.003) and distance (*p* < 0.001) fixation, divergence was better for near (*p* = 0.021) and distance (*p* < 0.001) fixation, accommodation was better in the right (*p* = 0.011) and left (*p* = 0.004) eyes, and the gradient AC/A ratio was better at the third month after injection (*p* = 0.001). Stereoacuity was improved in 11 (50%), unchanged in 5 (22.73%) and decreased in 6 (27.27%) patients. The preinjection stereoacuity (*p* = 0.013, *r* = 0.522) and preinjection deviation for near (*p* = 0.015 r, = − 0.512) and distance (*p* = 0.009, *r* = − 0.541) were significantly associated with patient age.

**Conclusions:**

AACE is characterized by a high AC/A ratio and low accommodation. A single injection of BTXA is effective for AACE. Deviation, stereoacuity, and the therapeutic effect of BTXA may be correlated with patient age.

## Background

Acute acquired comitant esotropia (AACE), a rare form of strabismus, describes sudden-onset constant esotropia with an equal angle of each gaze and leads to diplopia [1]. AACE was divided into the following 3 main types by Burian and Miller based on their different characteristics and etiologies [2]. Type 1 (Swan type) has sudden onset due to interrupted fusion (monocular occlusion or vision loss). Type 2 (Franceschetti type) may be caused by physical or mental stress (but not paralysis) and is characterized by large deviation, potential binocular vision, a low refractive error, and a minimal accommodative element. Type 3 (Bielschowsky type) is only relevant to the myopia of − 5.0 D or more.

In increasing case reports, AACE has been reported to be related to the refractive error, decompensated esophoria, intracranial diseases (elevated intracranial pressure, brain glioma, thalamic or cerebellar tumor), and near work such as excessive smartphone use. However, these cases cannot be included in the Burian and Miller classification [3–10]. Some AACE cases were neuroimaging study-negative, systemic disease-negative, and without a history of neuro- or anesthesia-related medication use. Such AACE cases were associated only with refractive errors and binocular dysfunction, such as decompensated esophoria and high myopia, which were generally considered benign or functional [3, 4, 11, 12].

AACE causes uncomfortable diplopia and substantially affects the quality of life. Surgery, botulinum toxin type A (BTXA) injection, and wearing prism are current proposed as treatments for AACE. BTXA injection has the advantages of improving stereoacuity, maintaining ocular alignment, and reducing anesthesia and treatment costs in comparison with surgery [13–15]. BTXA injection has been shown to be effective for esotropia at a high rate. It is effective for several weeks, and is deemed to be an alternative treatment or an addition to traditional surgery [15–17]. However, no study has investigated the characteristics of binocular visual function (BVF) of AACE and changes in BVF after BTXA injections.

To explore the possible characteristics of BVF in functional AACE patients, we conducted this study to examine the clinical features of AACE without intracranial and neurological diseases and then evaluated the changes in BVF after a single injection of BTXA.

## Methods

### Study population

This retrospective study enrolled patients with AACE examined at the Eye Center of the Second Affiliated Hospital, School of Medicine, Zhejiang University, from 1st October 2018-31st May 2019.

The inclusion criteria were as follows: 1) sudden-onset constant esotropia with an equal angle of each gaze and diplopia, followed by a diagnosis of AACE; and 2) BTXA injection in the both medial rectus muscles.

The exclusion criteria were as follows: 1) a history of eye disease, monocular occlusion or vision loss, ocular surgery (except for refractive surgery), and ocular or head trauma; 2) systemic diseases, including intracranial disease, neurologic disease (all patients underwent a head computerized tomography examination and a neurological examination by neurologists before BTXA injection to rule out intracranial and neurological diseases), and diabetes mellitus; 3) a history of neuro- or anesthesia-related medication use; and 4) refusal to cooperate with a BVF examination or to comply with the follow-up.

### Measurements of deviation angle and binocular visual function

We collected a complete medical history and personal details, including age, gender, the duration of diplopia, and the duration of daily near work from each patient. The following pre- and postinjection parameters were measured and recorded: refractive error, best-corrected visual acuity (BCVA), stereoacuity with the circles test of the Randot Stereotest (Stereo Optical Co., Inc.), the horizontal angle of deviation, vergence, accommodation, and the gradient accommodative convergence (prism diopter, PD)/accommodation (diopter, D) ratio (AC/A).

The horizontal angle of deviation was measured with the prism and cover tests at 33 cm (near) and 6 m (distance) fixation. After deviation was offset and diplopia was overlapped with a prism, convergence and divergence were re-examined with the prism and cover tests at near and distance fixation. This was measured by recording the patient’s reports of a break to base-out and base-in prism. The vergence value was the sum of convergence and divergence. The accommodation was measured at near fixation with a minus lens. Patients were instructed to keep the target clear as the minus lens degree increased and to report when it first started to become defocused. When the target first started to become defocused, the value of the increased minus lens degree plus 3D was taken as a measure of accommodation. The AC/A ratio was measured with the prism and cover tests at near fixation and determined by a deviation angle with additional pairs of lenses (+ 1.00 and − 1.00 D) [18, 19]. For statistical purposes, stereoacuity was converted to the reciprocal after measurement.

### BTXA injection method

BTXA (Allergan, USA) injection into the both medial rectus muscle was performed after surface anesthesia of the conjunctiva by the same surgeon. The injection dose of BTXA was 3.5 units for patients with a deviation angle less than 25 PD and 4.0 units for an angle greater than 25 PD. To reduce leakage of BTXA, the surgeon fixed the target muscle with forceps, used a 30 gauge × 1/2 in. (0.3 mm × 13 mm) needle during injection, and held the needle for a few seconds before withdrawing it.

### Statistical analysis

Statistical analyses were performed with SPSS version 21.0 (SPSS, Inc., Chicago, IL, USA). Data pre- and postinjection were compared by the Wilcoxon signed-rank test. Spearman’s correlation coefficient was calculated to explore relationships between demographic characteristics and BVF. *P* <  0.05 was considered statistically significant.

## Results

Twenty-two AACE patients (13 males and 9 females) with a mean age of 26.32 ± 7.19 (range: 14–38) years and sudden-onset, progressive, intermittent, or persistent horizontal binocular diplopia for 3 months to 10 years were included in the study. The mean duration of daily near work was 12.84 ± 2.88 (range: 8–18) hours.

The mean spherical equivalent refractions were − 4.35 ± 2.18D in the right eye and − 4.14 ± 2.26D in the left eye. Each patient with refractive error wore full-corrective glasses before and after BTXA injection for a large proportion of the day, including work, study, and daily life. Binocular BCVA was 20/20 in all patients except one, whose binocular BCVA was 18/20. Eye movements in all directions were free. Ocular examinations and neurological examinations were normal in all patients. The follow-up duration was 3 to 4 months. Clinical characteristics can be found in the Table 1.

**Table 1:** Summary of Patient Data

Patient No.	Age	Daily Near Work (hour)	Spherical Equivalent (D)		Pupillary Distance (mm)	Reciprocal stereoacuity		Deviation (PD)				Convergence (PD)				Divergence (PD)				Vergence (PD)				Accommodation (D)				Gradient AC/A (PD/D)	
			OD	OS		Pre- BTXA	Final	Pre-BTXA Distance	Final Distance	Pre-BTXA Near	Final Near	Pre-BTXA Distance	Final Distance	Pre-BTXA Near	Final Near	Pre-BTXA Distance	Final Distance	Pre-BTXA Near	Final Near	Pre-BTXA Distance	Final Distance	Pre-BTXA Near	Final Near	Pre-BTXA OD	Final OD	Pre-BTXA OS	Final OS	Pre-BTXA	Final
1	32	8	-3.375	-3.125	59	0.014	0.014	10	0	20	6	9	12	10	25	7	8	7	10	16	20	17	35	2.25	7	2.5	6.75	9	10
2	35	10	-5.25	-4.5	57	0.014	0.014	25	16	24	20	22	20	14	40	12	20	6	25	34	40	20	65	3.75	7.25	3.25	7.75	5	3
3	32	13	-4.25	-4	58	0.014	0.014	6	1	6	0.5	16	40	20	25	10	15	10	5	26	55	30	30	10	15.5	9	16.5	9	7
4	17	11	-4.25	-4.675	57	0.005	0.014	40	20	45	20	14	40	14	30	8	8	1	14	22	48	15	44	8.5	16.25	9	18	9	10
5	16	9	-6	-6.25	59	0.000	0.007	35	20	40	20	30	40	35	34	12	12	12	16	42	52	47	50	2	19	1.5	19.5	14	5
6	35	12	-0.25	-1	66	0.033	0.003	28	20	20	10	3	20	30	30	16	20	18	20	19	40	48	50	6.5	8.25	6.25	9	5	3
7	14	9	-3.75	-4	59	0.000	0.014	40	14	45	16	14	18	14	25	8	12	1	16	22	30	15	41	1	7.5	1	5.25	8	4
8	29	14	-4	-3.75	56	0.014	0.033	12	4	10	0	25	30	18	43	4	6	6	6	29	36	24	49	11	11.75	11	12	13	7
9	28	16	-7.875	-8.25	63	0.025	0.010	30	25	30	30	14	14	35	16	2	12	20	12	16	26	55	28	4.75	6.75	4	9.25	5	3
10	31	18	-7.75	-7.75	66	0.010	0.014	25	6	25	12	16	20	30	40	12	20	16	35	28	40	46	75	8.75	12.5	8.25	12.5	10	4
11	19	13	-7	-6	61	0.000	0.005	45	20	40	25	4	30	16	45	5	16	1	16	9	46	17	61	8.75	12.5	8.5	13	10	8
12	37	17	-4.75	-3.5	61	0.000	0.014	20	8	30	12	18	40	40	50	8	17	10	7	26	57	50	57	6.75	10.25	8	11	6	4
13	25	14	-3.75	-4	65	0.033	0.014	12	5	18	0	14	15	18	18	2	6	7	3	16	21	25	21	13.25	8.5	13.75	8	7	5
14	26	14	0	0	63	0.014	0.014	25	14	20	14	8	16	18	20	4	8	5	5	12	24	23	25	7.5	8.25	7.5	7	5	3
15	27	15	-4.125	-3.5	58	0.007	0.014	20	6	20	7	6	25	8	35	6	16	6	10	12	41	14	45	5	10.5	4.5	10.25	5	5
16	21	12	-4.25	-4.75	59	0.007	0.007	18	3	20	5	18	20	30	20	8	6	6	7	26	26	36	27	11.75	10.5	11.5	10.75	2	2
17	38	13	-4.25	-3.75	58	0.014	0.007	20	16	20	16	10	14	18	20	4	12	8	18	14	26	26	38	6	5.75	6	5.5	5	7
18	27	18	0	0	66	0.000	0.010	16	4	25	3	0.5	8	9	10	3	8	9	8	3.5	16	18	18	4.25	5.5	4.5	6.25	7	3
19	23	9	-5.25	-1.5	57	0.014	0.007	45	14	45	14	40	45	40	45	14	12	8	14	54	57	48	59	13	10.25	7.75	10	10	4
20	23	14	-3.75	-6.75	58	0.000	0.003	25	7	27	1	16	20	12	25	5	8	6	8	21	28	18	33	11.75	9.75	13.75	10.75	5	5
21	15	11	-4.875	-3.125	57	0.000	0.005	30	14	35	16	12	35	20	40	5	5	10	12	17	40	30	52	9.25	13.75	10.25	14.5	10	8
22	29	13	-7	-6.875	59	0.040	0.014	25	12	45	14	25	20	20	30	5	6	6	10	30	26	26	40	11.75	9.75	10.5	9.5	5	6

*BTXA* Botulinum toxin type A, *Final* The last follow-up at the 3 months after injection, *OD* Oculus dexter, *OS* Oculus sinister, *PD* Prism diopters, *D* Diopters. Patient 4,5,9,11,14,21 and 22 had persistent diplopia at 3 months after injection

Compared with preinjection deviation, the postinjection deviation in the primary position was smaller both for near (*p* < 0.001) and distance (*p* < 0.001) fixation at 3 months after injection. The divergence and convergence were better for both near and distance fixation. The accommodation was better in both eyes. The gradient AC/A ratio was better in the third month (Table 2). Stereoacuity was improved in 11 (50%), unchanged in 5 (22.73%) and decreased in 6 (27.27%) patients. All 7 patients without stereoacuity achieved stereoscopic vision after injection.

**Table 2:** Clinical characteristics of patients pre- and post-BTXA injection

Characteristics *n* = 22		Mean ± 1 Standard deviation (Range)				*P* Value
		Pre-BTXA injection		Post-BTXA injection		
Reciprocal stereoacuity		0.012 ± 0.012	(0.000–0.040)	0.012 ± 0.007	(0.003–0.033)	0.887
Deviation (PD)	Distance	25.09 ± 10.99	(6.0–45.0)	11.32 ± 7.23PD	(0.0–25.0)	< 0.001
	Near	27.73 ± 11.60	(6.0–45.0)	11.89 ± 8.40	(0.0–30.0)	< 0.001
Convergence (PD)	Distance	15.20 ± 9.25	(0.5–40.0)	24.64 ± 10.97	(8.0–45.0)	< 0.001
	Near	21.32 ± 9.95	(8.0–40.0)	30.27 ± 10.91	(10.0–50.0)	0.003
Divergence (PD)	Distance	7.24 ± 3.94	(2.0–16.0)	11.50 ± 5.00	(5.0–20.0)	< 0.001
	Near	8.14 ± 4.97	(1.0–20.0)	12.59 ± 7.42	(3.0–35.0)	0.021
Vergence (PD)	Distance	22.48 ± 11.29	(3.5–54.0)	36.14 ± 12.64	(16.0–57.0)	< 0.001
	Near	29.45 ± 13.49	(14.0–55.0)	42.86 ± 15.10	(18.0–75.0)	0.003
Accommodation (D)	OD	7.61 ± 3.69	(1.00–13.25)	10.32 ± 3.51	(5.00–19.00)	0.011
	OS	7.38 ± 3.68	(1.00–13.75)	10.59 ± 3.89	(5.25–19.50)	0.004
Gradient AC/A (PD/D)		7.45 ± 2.99	(2.0–14.0)	5.27 ± 2.31	(2.0–10.0)	0.001

*BTXA* Botulinum toxin type A, *Final* the last follow-up at the 3 months after injection, *OD* Oculus dexter, *OS* Oculus sinister, *PD* Prism diopters, *D* Diopters

Immediately following BTXA injection, diplopia disappeared in all patients. In the third month of follow-up, diplopia had disappeared or had appeared occasionally in 15 (68.2%) patients. 7 (31.8%) patients had persistent diplopia. None of the patients had complications such as infection, bleeding, ptosis, or restricted eye movement. No patients experienced overcorrection.

The Spearman’s correlation was calculated on demographic characteristics, deviation and BVF. The preinjection stereoacuity was not significantly associated with deviation for near (*p* = 0.066, *r* = − 0.399) and distance (*p* = 0.270, *r* = − 0.224), accommodation in the right eye (*p* = 0.267, *r* = 0.247) and left eye (*p* = 0.683, *r* = 0.092), and the duration of daily near work (*p* = 0.980, *r* = 0.006), but was associated with patient age (*p* = 0.013, *r* = 0.522). Preinjection deviations for near (*p* = 0.015, *r* = − 0.512) and distance (*p* = 0.009, *r* = − 0.541), and reductions in deviations after injection for near (*p* = 0.003, *r* = 0.601) and distance (*p* < 0.001, *r* = 0.699) were significantly associated with patient age. However, the duration of daily near work was not significantly associated with any BVF measured in this study.

## Discussion

The study showed that AACE was characterized by a high AC/A and low accommodation. A single injection of BTXA was effective for AACE. These findings may provide a reference for subsequent treatment.

AACE has long been divided into 3 main types based on the characteristics of interrupted fusion, physical or mental stress, and mildly hypermetropia [2]. However, some reported cases did not fit into these types [3–5, 7, 8, 12]. According to imaging evidence, neurological and intracranial diseases are undoubtedly the causes of AACE [5, 6, 9, 10]. In this study, the AACE patients did not suffer from intracranial or neurological diseases. Some patients suffered from high myopia or esotropia with a small angle. Without a history of eye disease, monocular occlusion or vision loss, ocular surgery, or ocular trauma, the patients in this study did not meet the classification. A new classification should be proposed to cover these cases. Therefore, functional or benign causes of AACE should be considered, and BVF should be examined.

After excluding diseases of the brain, extraocular muscles, and orbit, Ali et al. included 8 cases of progressive intermittent horizontal esotropia and speculated that decompensated esophoria was a benign cause of AACE [4]. However, their study did not study for AC/A ratio. The AC/A ratio shows some characteristics in comitant strabismus and changes after treatment. Bateman and Parks found that the AC/A was normal, high, and low in 62, 34, and 4% of concomitant esotropia patients, respectively. AC/A ratio was closer to normal after surgery [20]. A prospective study by Lucas et al. found the mean AC/A of 25 patients with comitant esotropia decreased from 8 to 4 [21]. In this study, the mean AC/A ratio of 22 patients was higher than those in normal (between 3 and 5 PD/D) [19] and esotropic (an average of 3.15 PD/D) populations [22], and accommodation was lower than age-matched values [23]. The AC/A ratio was also found to be high in convergence excess cases [24], suggesting that convergence excess may be a characteristic of AACE, which produces more convergence with the same accommodation requirements [19]. The lack of accommodative ability leads to more convergent accommodation when patients view objects at close range. This induces chronic overuse of the both medial rectus muscles in patients with AACE, which may strengthen the medial rectus muscles and break the balance of the medial and lateral rectus muscles. This may subsequently be the cause of AACE in patients using smartphones at close proximity for a long time [7, 8].

Refractive error may be another cause of AACE. In a study by Simon and Borchert [3], after correcting for hypermetropia, esotropia improved in seven patients. The study speculated that an uncorrected refractive error may be the cause of acute late onset esotropia. As each patient with refractive error wore full-corrective glasses for most of their daily lives in our study, we found that preinjection deviation for near and distance was not associated with the spherical equivalent in each eye. This may partly support Simon and Borchert’s view and suggest that correction of refractive errors is not correlated with the incidence of AACE. This hypothesis should be tested in a controlled clinical trial.

BTXA is a neurotoxin that selectively blocks the release of acetylcholine at the neuromuscular junction and inhibits muscle contraction [25]. Therefore, BTXA affects BVF associated with the injected extraocular muscles. However, few studies have described BVF changes postinjection. After a single BTXA injection in this study, the deviation of the AACE patients decreased. The convergence, divergence, and accommodation improved. The post-injection AC/A ratio in this study decreased and was close to normal, which may suggest that BTXA can relieve excessive convergence by inhibiting contraction of the medial rectus muscle. The accommodation in our study was reflex accommodation induced by a blurred retinal image with a minus lens. We could not find conclusive evidence to support the effect of a high AC/A ratio or excessive convergence on reflex accommodation in existing studies. However, we can speculate that the increase in convergence accommodation may lead to a decrease in reflex accommodation, when the amplitude of accommodation is fixed. Accommodation improved at 3 months after the injection, which may have been due to remission of convergence accommodation after deviation was alleviated.

Compared with globe perforation, slipped muscle, scleritis, and intraoperative muscle loss, BTXA is less invasive, muscle-sparing [26], and the injection repeatable up to 9 times with no significant side effects [15]. Without the need for incision or suturing of the extraocular muscles and scar formation, patients may achieve closer to normal BVF after BTXA injection. Based on this speculation, we will set up a surgery group and a BTXA injection group for comparison in further study.

BVF change with age. Older patients (40–70 years of age) had a more tonic convergence position than younger patients (20–39 years of age) [27]. The magnitude of phoria adaptation and the base-in and base-out recovery decreased with age [28, 29]. The AC/A ratio increases with age, which may be associated with tonic accommodation and vergence systems [30]. Therefore, we also considered the effect of age on the visual factors of AACE. Research on the epidemiology of AACE is currently lacking. In existing studies and case reports, most AACE patients are children and young adults. Fu et al. found that the mean deviation of esotropia in younger children was significantly larger than that in older children and adults in the Chinese population. A younger onset age may be a characteristic of AACE and may also be associated with esotropia deviation [11]. In our study, the younger patients experienced worse preinjection stereopsis and larger preinjection deviations for near and distance. We suspect that the stronger esophoria adaptation and recovery of younger patients delayed the onset of diplopia, leading to a more advanced stage of AACE and a greater deviation angle. The reductions in deviations for near and distance in this study were greater in older patients, possibly because the BTXA injection attenuated more tonic convergence in older patients when inhibiting muscle contraction.

Uncorrected strabismus or the presence of diplopia after treatment is defined as recurrence. Clark et al. reported a 1/6 (16.7%) recurrence rate for AACE treated by surgery [1]. Dawson et al. reported that 2/14 (14.3%) patients had unstable ocular positions after toxin injection [13]. In a study conducted by Wan et al., the success rates of BTXA injections were 81% at 6 months and 67% at 18 months, compared with the surgery group, where the success rates were 61% at 6 months and 58% at 18 months [14]. Conversely, Alejandra et al. reported a treatment success rate of 66% of surgical procedures compared with 45% of BTXA injections [16]. In this study, although deviation was large in some patients, they had similar or greater divergent functions numerically. These patients experienced diplopia only in the morning or with severe eye strain. In contrast, some patients had less deviation but substantially less divergence, with persistent diplopia after the injection. Before this study, AACE patients showed symptoms of overcorrection after BXTA injection. We gradually adjusted the dose and determined the current injection dose of 3.5–4 units. In this study, no patients experienced overcorrection.

Since the dose of BTXA cannot be accurately measured according to the angle of deviation and the main objective of AACE treatment is diplopia relief, BTXA treatment of AACE can be considered satisfactory under the following conditions: 1) divergence ability after treatment can compensate for strabismus; 2) diplopia symptoms do not appear in daily life; 3) a satisfactory quality of life can be achieved. In our study, diplopia disappeared or appeared only briefly in 15 (68.2%) patients after a single BTXA injection, reflecting a satisfactory therapeutic effect. Patients with recurrence can still be reinjected with BTXA or treated with surgery.

Some limitations may exist in this study. AACE patients were in a state of binocular diplopia and esotropia when they visited the doctor. BVF before onset was unknown and difficult to assess. Secondly, the dominant eye may have switched due to diplopia and BVF changed. The possible influence of dominant eye function on AACE was difficult to investigate. Finally, the follow-up period was short, and the long-term recurrence of AACE after a single BTXA injection was unknown.

## Conclusion

AACE without intracranial and neurological diseases is characterized by a high AC/A and low accommodation. A single injection of BTXA is effective for AACE. Patient age may be an important factor correlated with deviation, stereoacuity, and the therapeutic effect of BTXA.

## Data Availability

The datasets used and analyzed during the current study are available from the corresponding author on reasonable request.

## Abbreviations

AACE: Acute acquired comitant esotropia
BTXA: Botulinum toxin type A
BVF: Binocular visual function
D: Diopter
PD: Prism diopter
BCVA: Best-corrected visual acuity
AC/A: Accommodative convergence/accommodation ratio
